# Predictors of Developmental Patterns of Obesity in Young Children

**DOI:** 10.3389/fped.2020.00109

**Published:** 2020-03-24

**Authors:** Thomas G. O'Connor, Jason Williams, Clancy Blair, Lisa M. Gatzke-Kopp, Lori Francis, Michael T. Willoughby

**Affiliations:** ^1^Department of Psychiatry, University of Rochester, Rochester, NY, United States; ^2^Department of Psychology, University of Rochester, Rochester, NY, United States; ^3^Department of Neuroscience, University of Rochester, Rochester, NY, United States; ^4^Department of Obstetrics and Gynecology, University of Rochester, Rochester, NY, United States; ^5^Wynne Center for Family Research, University of Rochester, Rochester, NY, United States; ^6^RTI International, Research Triangle Park, Durham, NC, United States; ^7^Department of Population Health, New York University, New York, NY, United States; ^8^Department of Human Development and Family Studies, The Pennsylvania State University, State College, PA, United States; ^9^Department of Biobehavioral Health, The Pennsylvania State University, State College, PA, United States

**Keywords:** obesity, birth weight, socio-economic status, developmental programming, social determinants

## Abstract

**Introduction:** The current study characterizes longitudinal patterns in obesity in young children and their prediction from developmental programming and social determinant hypotheses.

**Materials and Methods:** The data are based on the Family Life Project, a prospective longitudinal study of 1,292 families recruited from low-income, racially diverse, rural communities in Pennsylvania, and North Carolina. Pre-natal, peri-natal, and post-natal risks for childhood obesity were collected from 2 months of age; in-person assessments of child growth were used to identity obesity on multiple occasions from 24 to 90 months of age.

**Results:** Two major novel findings emerged. First, longitudinal analyses identified four distinct obesity development profiles: stable obesity, later-onset obesity, moderate/declining obesity, and non-obese; these groups had distinct risk profiles. Second, prediction analyses favored developmental programming explanations for obesity, including evidence even in early childhood that both low- and high birth weight was associated with stable obesity. There was no indication that pre- and peri-natal and post-natal factors predicted obesity differently in non-minority and minority children.

**Discussion:** Factors derived from the developmental programming model of obesity overlapped with, but predicted early onset obesity independently from, risks associated with social determinant models of obesity.

## Introduction

Childhood obesity is a recognized public health concern because of its prevalence, persistence, and link with current and future health problems (1–6). A major focus of research is the identification of early modifiable risks that could improve clinical assessment, intervention, and prevention. The highly influential developmental programming hypothesis focuses on early exposures, including those *in utero*, that may set or “program” metabolic processes (7–9). These early exposures may have persisting influence on health outcomes to the extent that they induce early and persisting metabolic adaptations that are poorly suited to later environmental conditions (10). Specifically, pre-natal exposures such as maternal obesity and diet may shape the developing child's metabolic processes—and therefore later obesity risk—because they signal something about the current and future nutritional environment (11–14). In addition, some evidence suggests that rapid post-natal weight gain of the child is associated with later obesity (15–18); one possible explanation is that this rapid weight gain may alter child metabolism leading to increased obesity risk. Low birth weight has a prominent role in the developmental programming literature because it is hypothesized to reflect a poor pre-natal nutritional environment. Indeed, a widely-replicated finding, and the basis for the “thrifty phenotype” hypothesis in the developmental programming literature (19), is that low birth weight is associated with adult cardio-metabolic disease (7, 20–22). It is not yet clear if, as suggested by the thrifty phenotype hypothesis, low birth weight is a risk for early-onset obesity. To date, studies of pediatric and adult samples indicate that high birth weight as a predictor of obesity (23, 24). We extend current research to consider the key developmental hypothesis that early-onset obesity is also associated with low birth weight.

Largely separate from the above studies on the developmental programming hypothesis is research seeking to identify social determinants of childhood obesity. Social determinants hypotheses highlight the predictive role of low socio-economic status (1, 4, 25–28), child health behaviors, and race/ethnicity and culture (29–31) rather than pre- and peri-natal risks. The social determinants framework contrasts sharply with the developmental programming framework for childhood obesity: they propose different mechanisms, imply different interventions and different timing for these interventions. The current study was designed to contrast these two alternative hypotheses for early onset obesity.

We first consider the potential confounding of predictors from each model, a significant methodological and conceptual matter afforded little attention to date. Some evidence of confounding of these models is suggested. For example, bottle feeding is associated with rapid early weight gain (32) and low socio-economic standing (33), and may offer one example of how developmental programming and social determinant models may be confounded. In addition, low birth weight is a target for the developmental programming models of obesity but it is also associated with social determinants model, in terms of socioeconomic status (34) and race (35–37). Furthermore, if there is a greater risk of low birthweight in African-American infants, then this may explain race differences in early-onset obesity. More broadly, this line of investigation suggests that developmental programming risks may be moderated by social context. We examine this novel hypothesis in the current study.

An important observation from longitudinal studies of obesity is the significant within-person change (38–40), even in infancy (41). The implication is that longitudinal data are needed and that analyses must consider not a singular time point but rather a developmental profile that reflects within-person change in obesity status. However, to date, most studies rely on a single measurement occasion and so miss important within-person change. A key feature of the current study is that we adopt a developmental approach to defining early obesity status by assessing longitudinal patterns of obesity based on approximately 6 years of data from multiple occasions of measurement. We do this in an epidemiologically-derived sample of 1,292 low-income families with a high rate of minority participation.

## Materials and Methods

### Study Population

The Family Life Project (FLP) is an ongoing longitudinal study of rural poverty that involves families who delivered a child between September 2003-August 2004 in one of six counties in Eastern North Carolina (NC) and Central Pennsylvania (PA). Complex sampling procedures were employed to recruit a representative sample of 1,292 children from rural counties, with over-sampling of low-income families in both states and of African American families in NC (42). The current study makes use of data that were collected in home visits when children were 2, 6, 15, 24, 35, 48, and 58 and 90 months of age. This study is limited to 1,164 children for whom at least one measure of BMI was available at the 24-, 35-, 48-, 58-, or 90 months home visits. The study was approved by the local IRB; written consent was obtained; families were compensated for participation.

### Procedures

Following hospital screening, participants who were selected and agreed to participate were formally enrolled into the study by completing a home visit when the target child was approximately 2 months old. BMI values from age 24 through 90 months were used to index the outcome variable, developmental trajectories of obesity. Maternal report and physical measurements from the 2, 6, 15, and 24 months home visits were used to construct the focal predictors from age 24 through 90 months.

### Measures

#### Socioeconomic Status and Socio-Demographics

SES was included in the study as a latent factor score based on a confirmatory factor analysis of four items. The first is the highest level of education for the primary caregiver: less than high school degree (10%), high school graduate (69%), bachelor's degree or higher (21%); a dummy code was created to indicate whether or not the caregiver completed high school. The second indicator was household poverty, which was calculated by summing the total household income by the federal poverty threshold for a given family size to create the income/needs ratio (INR) at the 6 months visit. The third indicator was receipt of income-based government assistance for nutrition (Special Supplemental Nutrition Program for Women, Infants, and Children) at the 2 months visit. The fourth indicator was the presence of a spouse or partner in the home at the 2 months visit. Parent-reported child race (African-American or Caucasian) was included as an additional socio-demographic predictor.

#### Pre- and Peri-Natal Risks

At the 2 months home visit, mothers self-reported their pre-pregnancy weight in pounds, which was used in conjunction with their measured height to calculate pre-pregnancy body mass index (BMI). Infant's birth weight was reported by mothers; this was validated in a subset of cases in which we also obtained birth weight from medical birth records [*r*_(150)_ = 0.92; ICC = 0.90]. Infant gestational age was based on maternal reports. Infant early weight gain (birth to 24 months) was constructed from birth weight and measured weight collected by researchers in the home to construct an average monthly gain variable (kg/month units). Pre-natal smoking was based on maternal report; given the limited range, this was re-scaled as yes/no. Breast-feeding was scaled to indicate any breastfeeding through 6 months of age.

#### Child BMI and Obesity

Children's standing height and weight at the 24-, 35-, 48-, 58-, and 90 months home visits was measured by trained researchers, and was used to generate age- and sex-specific body mass indices using the 2000 CDC growth charts (http://www.cdc.gov/growthcharts/). At each of the five assessments, BMI percentile values ≥95% were identified and used to define obesity status (the Statistical Analyses section describes how these 5 time points were combined to create distinct developmental trajectories or latent classes).

### Statistical Analyses

Descriptive statistics and bivariate analyses are first reported. Obesity class was based on a series of latent class analyses of the five occasions of measurement of obesity status from 24 to 90 months. The best fitting class was established by comparison of the sample size adjusted Bayesian Information Criterion (BIC) and the Vuong-Lo-Mendell-Rubin likelihood ratio test for a series of models that extracted a range of classes from the data (43). Once the best fitting latent class model was identified, predictors were introduced to predict latent class membership. Predictive models used the 3-step method which assumes that class membership is probabilistic (44). Predictor variables are included in the final model that make an independent prediction or substantively alter other parameter estimates in the model; race was considered as a moderator.

## Results

### Preliminary Analyses and Descriptive Statistics

The 1,164 participating children with BMI data did not differ from the 128 non-included children with respect to state of residence (40.3 vs. 40.6% residing in PA, *p* = 0.40), income to needs ratio (77.8 vs. 74.8% poor, *p* = 0.46), primary caregiver educational status at study enrollment (80.4 vs. 79.7% with a high school degree or GED, *p* = 0.84), sex of the child (50.5 vs. 54.5% male, *p* = 0.40), race of the child (43.1 vs. 35.0% African American, *p* = 0.08). Descriptive data of the participants are provided in Table 1.

**Table 1:** Descriptive statistics for overall sample and by each obesity development profile.

	**Overall *N* = 1,164**	**Stable obesity *N* = 90**	**Later-onset obesity *N* = 74**	**Low obesity *N* = 922**	**Moderate/declining obesity *N* = 78**
Non-white (%)	0.43	0.48	0.45	0.43	0.40
Breast fed until 6 mos (%)	0.14	0.06	0.07	0.15	0.14
Maternal pre-pregnancy BMI (M/SD)	26.47 (0.22)	30.4 (0.9)	30.01 (1)	25.82 (0.23)	26.68 (0.9)
Gestational age (weeks) (M/SD)	38.68 (0.06)	38.71 (0.2)	38.96 (0.2)	38.67 (0.06)	38.48 (0.28)
SES (M/SD)	−0.21 (0.02)	−0.16 (0.1)	−0.16 (0.08)	−0.21 (0.02)	−0.22 (0.08)
Weight gain (g) (M/SD)	0.7 (0.00)	0.79 (0.01)	0.72 (0.02)	0.69 (0.00)	0.76 (0.02)
Birth weight (g) (M/SD)	3274.52 (17.21)	3411.39 (71.89)	3374.36 (70.2)	3239.75 (18.43)	3432.84 (80.28)
Smoked during pregnancy (%)	0.23	0.27	0.31	0.22	0.29
Obesity, 24 mos	0.13 (0.01)	0.72 (0.06)	0.11 (0.04)	0.04 (0.01)	0.5 (0.08)
Obesity, 35 mos	0.11 (0.01)	1 (0)	0 (0)	0 (0)	0.48 (0.06)
Obesity, 48 mos	0.14 (0.01)	0.95 (0.03)	0.71 (0.05)	0 (0)	0.33 (0.06)
Obesity, 58 mos	0.17 (0.01)	0.98 (0.02)	0.93 (0.03)	0 (0)	0.41 (0.06)
Obesity, 90 mos	0.17 (0.01)	0.89 (0.04)	0.85 (0.04)	0.05 (0.01)	0.05 (0.03)

*Values of continuous variables are shown with mean (M) and standard deviation (SD); for categorical (in all cases dichotomous) variables, the percent affected is provided. Obesity rates in the whole sample at each assessments are provided toward the bottom of column 1 with the SE, e.g., the rate of obesity in the whole sample was 17% at 58 months and 17% at 90 months of age. Corresponding values for the 4 classes (columns 2–5) represent the likelihood of obesity for a given child in that group with its SE, e.g., at 90 months, the likelihood that a child in the Stable obesity group was obese was 89%; the likelihood that a child in the Later-onset Obesity group was obese was 85%; the likelihood that a child in the Low obese or Moderate/Declining group was obese was just 5%*.

### Obesity Risk Status

**Figure 1:**
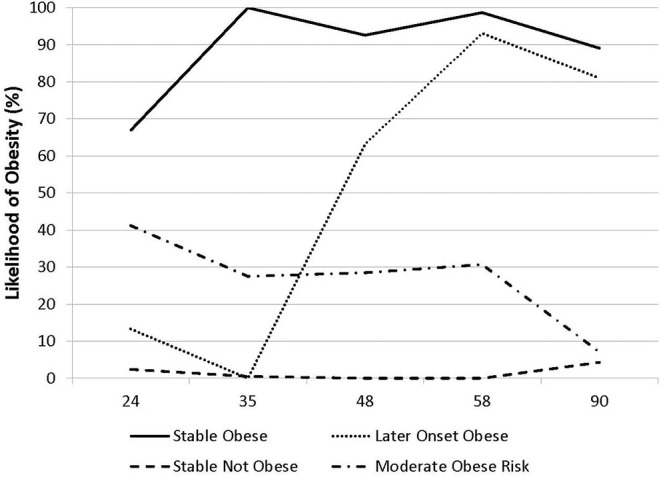
Likelihood of obesity by LCA class (by child age in months).

Year-by-year rates of obesity varied from 13% at 24 months to 17% at 90 months. We found no evidence of very low weight children (<5%) from age 2 years; as a result, all analyses target obesity. Figure 1 shows the results from the latent class analysis using obesity status at the 5 occasions of measurement. Four groups of children were identified that optimized the model fit. The largest group (*n* = 922; 79%; Stable Not Obese) is a stable low obesity group; a stable obesity group composed 8% of the sample (*n* =90; Stable Obesity); two other groups were identified: a later-onset obesity group (*n* = 74; 6%; Later-onset Obesity) which exhibited a marked increase in obesity status over the assessment period and a moderate and declining group (*n* = 78; 7%; Moderate-declining Obesity) which exhibited low obesity by the final assessment. These four groups form the basis for prediction analyses described below.

### Bivariate Associations Among Predictors

Associations between predictors are provided in Table 2. The results imply a notable degree of overlap between variables associated with social determinant and developmental programming models.

**Table 2:** Correlations among key predictor variables.

	**Maternal pre-pregnancy BMI**	**Smoked during pregnancy**	**Birth weight (g)**	**Gestational age (weeks)**	**Weight gain (kg)**	**Breast fed until 6 mos**	**Non- white**
SES	−0.07^*^	−0.15^***^	0.15^***^	−0.027	0.02	0.33^***^	−0.42^***^
Maternal pre-pregnancy BMI		−0.07^*^	0.06^*^	−0.076^*^	−0.03	−0.05^*^	0.15^***^
Smoked during pregnancy			−0.16^***^	0.02	0.06	−0.12^***^	−0.13^***^
Birth weight (g)				0.58^***^	−0.13^***^	0.09^**^	−0.18^***^
Gestational age (weeks)					−0.14^***^	0.02	−0.04
Weight gain (kg/month)						−0.11^***^	−0.02
Breast fed until 6 mos							−0.24^***^

* *p < 0.05*,

** *p < 0.01*,

*** *p < 0.001*.

### Predicting Child Obesity Class Membership

**Figure 2:**
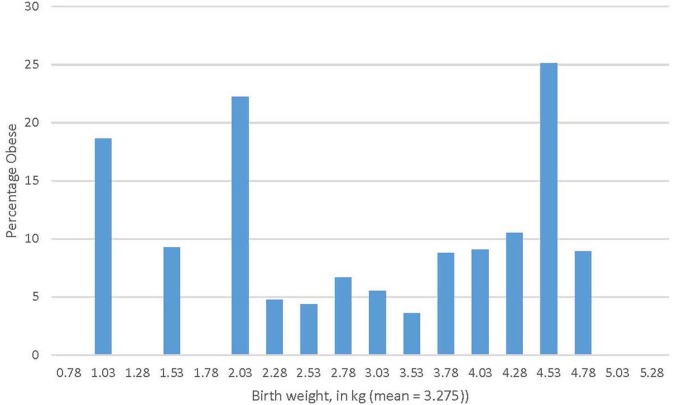
Non-linear association between birth weight and subsequent early-onset obesity.

Results from a regression model predicting obesity class membership are provided in Table 3; the class of Stable Not Obese children is the reference group, i.e., analyses in Table 3 indicate the effect of a target group compared with the Stable Not Obese group. As column 1 in Table 3 indicates, compared to children in the Stable Not Obese group, children in the Stable Obesity class were comparatively more likely to have a mother with elevated pre-pregnancy BMI who smoked in pregnancy; children in the stable obesity group also exhibited a rapid early weight gain. In addition, children in the Stable Obesity class also had a significantly greater weight at delivery but, notably, there was a quadratic effect of birth weight, which is shown in Figure 2: both low birth weight *and* high birth weight differentiated children who showed early (i.e., from 2 years) and stable obesity from children who were never obese.

**Table 3:** Prediction analyses differentiating children in obesity classes from children in the non-obese group: Odds Ratios.

	**OR (95% confidence interval)**		
	**Stable obesity**	**Later-onset obesity**	**Moderate-declining obesity**
Non-white	2.05 (0.93–4.55)	1.06 (0.47–2.36)	1.28 (0.47–3.53)
Breast fed until 6 mos	0.47 (0.16–1.4)	0.42 (0.07–2.38)	1.6 (0.58–4.39)
Maternal pre-pregnancy BMI	1.10^***^ (1.06–1.14)	1.11^***^ (1.07–1.16)	1.03 (0.97–1.10)
Gestational age	1.05 (0.81–1.37)	1.01 (0.70–1.47)	0.76 (0.58–1.01)
SES	1.37 (0.86–2.18)	1.34 (0.93–1.93)	0.82 (0.48–1.39)
Weight gain (grams/month)	1.01^***^ (1.01–1.01)	1.00 (0.99–1.01)	1.01^**^ (1.00–1.01)
Smoked during pregnancy	2.95^**^ (1.37–6.38)	2.81^*^ (1.14–6.90)	1.86 (0.72–4.85)
Birth weight (grams)	1.72^*^ (1.12–2.63)	1.32 (0.71–2.43)	2.95^***^ (1.74–5.00)
Birth weight (grams), squared	1.23^*^ (1.05–1.44)	1.08 (0.85–1.37)	1.07 (0.83–1.37)

*The reference group for each analysis is the Stable Not Obese group; each column represents a different contrast (from one simultaneous analysis). Results in Column 1 identify factors differentiating children in the Stable Obesity group from the Not Obese group; results in Column 2 identify factors differentiating children in the Later-onset Obesity group from the Not Obese group; results in Column 3 identify factors differentiating children in the Moderate-declining Obesity group from the Not Obese group. See Figure 1 for a description of the 4 groups of children identified from the latent class analysis*.

* *p < 0.05*,

** *p < 0.01*,

*** *p < 0.001*.

Latent class analysis identified two other classes of children that were comparable in size to the class identified by Stable Obesity but with markedly different developmental patterns of obesity from 24 to 90 months of age (Figure 1). Importantly, children with these other developmental patterns of obesity could also be differentiated from those children in the Stable Not Obese group, and they were rather different from the children in the Stable Obesity class. Specifically, children who displayed a “Later-onset” Obesity pattern were reliably different from children in the Stable Not Obese group only in terms of maternal pre-pregnancy BMI and maternal pre-natal smoking (Table 3). A further class of children who exhibited a Moderate-declining Obesity pattern were distinguished from children in the Stable Not Obese group only in having a larger birth weight (Table 3); it would seem that the apparent transient and modest increase in obesity was explained only by higher birth weight.

Additional analyses indicated that the prediction of obesity from individual risks or to latent class groups did not differ by race or social class. That is, for example, race did not moderate the risk of pre-, peri-, or early post-natal risks on obesity in early childhood (results not tabled).

### Supplementary and Sensitivity Analyses

A series of additional analyses were conducted to examine the robustness of the prediction analyses. One novel finding was the linear and quadratic effect of birth weight (measured as a continuous variable) that differentiated children in the stable obese group from children in the stable non-obese group. Additional analyses indicate that the quadratic effect did not derive from clinical low birth weight (<2,500 g), which was not associated with obesity status risk or obesity class. Second, analyses presented here were not substantially affected by adjusting for gestational age at birth, consistent with other reports (45).

## Discussion

Identification of obesity by age 6 years was recently promoted by the U.S. Preventive Services Task Force (USPSTF) (46) given the finding that early-onset obesity poses particular clinical and public health challenges. The current study provides valuable new findings on the predictors of obesity in early childhood from the Family Life Project, which incorporated several innovative features for contributing to the already sizable literature on childhood obesity. These include, most notably, an over-sampled low-income, rural population with a comparatively high concentration of African-American children, and a prospective longitudinal design that included frequent assessments in infancy and early childhood that offered particular power for tracking obesity—including within-person change in obesity status.

Results indicated that socio-economic status was associated with many pre-, peri-, and early post-natal predictors of obesity highlighted by a developmental programming model, but these socio-economic or social determinant risks did not independently predict early-onset obesity. A novel finding in this analysis was the detection of a curvilinear association between birth weight and the developmental profile of obesity status even in early childhood: stable obesity group membership in early childhood was associated with both higher birth weight and lower birth weight (this effect was limited to the subgroup of children who showed the most severe and persistent pattern of early obesity). The impact of higher birth weight on obesity is well-documented; the demonstration that low birth weight also forecasts later obesity is novel and implies the existence of a “thrifty phenotype” (19) in a subtype of children with stable early-onset obesity. Further follow-up of this sample will be needed to examine if the increased risk of obesity associated with high and low birthweight is accompanied by obesity-related markers of disease such as insulin resistance or high blood pressure.

A second novel finding, which was made possible by the intensive longitudinal assessment schedule, was the existence of several groups with distinguishable obesity patterns and predictors. Importantly, the patterns imply notable intra-individual changes in obesity even in early childhood—with distinct patterns of prior risk exposure compared to children at low risk of obesity (Figure 1 and Table 3). Specifically, alongside the stable obese group was a distinct and comparably sized group whose obesity was evident at the later but not earlier assessments. The later-onset obesity group of children exhibited a likelihood of obesity that was comparable to those children who had exhibited a high and earlier likelihood of obesity by age 2 years and did show the “classic” array of developmental programming risks. Further follow-up of these two distinct developmental trajectories, which is now underway, may reveal if there are notable differences in future health outcomes and biological markers of risk. Analyses allowing for within-individual change also identified a class of children with apparently transient obesity risk. This notable subset of children in the moderate and declining obesity class exhibited elevated birth weight (along with other obesity groups) but no lasting risk of obesity by middle childhood. Further follow-up of these children may also reveal that they do not show lasting risk of cardio-metabolic risk; that may explain why, for some children, high birth weight, and later (transient) obesity does not convey adverse health outcomes.

A third notable finding was the lack of independent effect of low SES or race on obesity classes in early childhood. Several papers based on the social determinant model indicate that social class factors are associated with childhood obesity in young children (4); therefore, a lack of an SES effect on obesity class or developmental pattern was somewhat unexpected. However, the degree to which findings from this study conflict with prior studies is not necessarily straight-forward (25). That is because prior studies demonstrating a role of social factors were based on a single time-point and, likely more importantly, did not consistently account for the programming-related factors such as pre-pregnancy BMI, low birth weight, and early weight gain. As our and others' findings show, many of these factors are confounded with social class (47). Alternatively, it may be that the lack of a reliable SES association with obesity may be a function of the concentration of low SES families or the young age of the children in the study; further follow-up may identify an emergent impact of SES-related influences on obesity that are separable from or compound the programming effects shown here.

There are several factors that may limit the generalizability of the findings. One is coverage of possible confounds and covariates, such as pre-natal maternal hypertension, hyperlipidemia, and diet; or, postnatally, information on young children's exercise and the built environment. At least some of these factors have not been found to associate with obesity risk in early childhood (4, 48, 49), but it remains possible that unmeasured variables confound our results. In addition, we did not have data on biological mechanisms associated with metabolic control of obesity, such as insulin resistance, at these early ages; however, obesity status is a notable outcome in early childhood because of its established links with later cardio-metabolic disease, [e.g., (6)]. Also, we had incomplete access to birth record data and so relied on maternal reports, and note the high agreement with medical record data. Furthermore, given that the study over-sampled low SES families, it may be that the resulting limited range (especially at the highest end) of SES may have artificially attenuated its association with pediatric obesity. Limitations of the study were offset by several strengths, including a comparatively large sample size; economic and racial diversity; availability of important covariates (maternal pre-pregnancy BMI, breastfeeding, early post-natal growth) that are often missing from other large-scale studies; and detailed exposure and growth data measured on multiple occasions.

Much has been written about the generally poor treatment outcomes for childhood obesity and the entrenched patterns of obesity that are evident by early childhood. Our findings extend and strengthen the emerging focus on early interventions because the risk mechanisms for obesity may be triggered by pre- and peri- and early post-natal exposures. More specifically, the current results imply that clinical impact may be optimized by focusing on early exposures, from obesity in pregnancy to growth patterns in the early months of life. That is emerging as a lesson from many studies; the current study extends that finding to the population at possible greatest risk of obesity but who nevertheless are under-represented in cohort studies—low-income, rural, minority families.

## Data Availability Statement

The datasets generated for this study are available on request to the corresponding author.

## Ethics Statement

The studies involving human participants were reviewed and approved by University of North Carolina IRB. Written informed consent to participate in this study was provided by the participants' legal guardian/next of kin.

## Author Contributions

TO'C conceived analyses, interpreted results, and drafted the paper. JW and MW conducted analyses and interpreted analyses. All authors were involved in interpreting the analyses and editing the paper and had final approval of the submitted and published versions.

### Conflict of Interest

The authors declare that the research was conducted in the absence of any commercial or financial relationships that could be construed as a potential conflict of interest.
